# When moving faces activate the house area: an fMRI study of object-file retrieval

**DOI:** 10.1186/1744-9081-4-50

**Published:** 2008-10-22

**Authors:** André W Keizer, Sander Nieuwenhuis, Lorenza S Colzato, Wouter Teeuwisse, Serge ARB Rombouts, Bernhard Hommel

**Affiliations:** 1 Institute for Psychological Research, Leiden University, Wassenaarseweg 52, 2300 RB Leiden, The Netherlands; 2 Leiden Institute for Brain and Cognition, Leiden University, Albinusdreef 2, 2300 RC Leiden, The Netherlands; 3 Department of Radiology, Leiden University Medical Center, Albinusdreef 2, 2300 RC Leiden, The Netherlands

## Abstract

**Background:**

The visual cortex of the human brain contains specialized modules for processing different visual features of an object. Confronted with multiple objects, the system needs to attribute the correct features to each object (often referred to as 'the binding problem'). The brain is assumed to integrate the features of perceived objects into object files – pointers to the neural representations of these features, which outlive the event they represent in order to maintain stable percepts of objects over time. It has been hypothesized that a new encounter with one of the previously bound features will reactivate the other features in the associated object file according to a kind of pattern-completion process.

**Methods:**

Fourteen healthy volunteers participated in an fMRI experiment and performed a task designed to measure the aftereffects of binding visual features (houses, faces, motion direction). On each trial, participants viewed a particular combination of features (S1) before carrying out a speeded choice response to a second combination of features (S2). Repetition and alternation of all three features was varied orthogonally.

**Results:**

The behavioral results showed the standard partial repetition costs: a reaction time increase when one feature was repeated and the other feature alternated between S1 and S2, as compared to complete repetitions or alternations of these features. Importantly, the fMRI results provided evidence that repeating motion direction reactivated the object that previously moved in the same direction. More specifically, perceiving a face moving in the same direction as a just-perceived house increased activation in the parahippocampal place area (PPA). A similar reactivation effect was not observed for faces in the fusiform face area (FFA). Individual differences in the size of the reactivation effects in the PPA and FFA showed a positive correlation with the corresponding partial repetition costs.

**Conclusion:**

Our study provides the first neural evidence that features are bound together on a single presentation and that reviewing one feature automatically reactivates the features that previously accompanied it.

## Background

The human visual cortex is divided into specialized modules that code a variety of different visual features, like motion in area MT/MST [1,2], faces in the fusiform face area (FFA; [3]) and houses in the parahippocampal place area (PPA; [4]). This division of labor entails a well-known problem: When confronted with multiple objects, how does the visual system 'know' which features belong together in one object?

This so-called 'binding problem' [5] calls for the integration of information into object representations or 'object files' [6]. The immediate consequences of such integration have been demonstrated in an elegant study by O'Craven et al. [7]. Their subjects saw overlapping pictures of a house and a face, with either the house or the face moving. When subjects were asked to respond to the direction of the motion, attention spread from the motion to the object, regardless of which object was moving: Functional magnetic resonance imaging (fMRI) results showed that the PPA was activated more strongly when the house moved, and the FFA was activated more strongly when the face moved. This suggests that attending to an event creates some sort of functional link between the representations of its features, whether they are relevant (like the direction of the motion in this example) or irrelevant (like the faces or houses). Further support for this notion comes from a recent fMRI study by Yi et al. [8] who found that face-selective regions in the FFA and lateral occipital cortex exhibited significantly less activation when (task-relevant) faces were repeated in (task-irrelevant) continuous versus discontinuous trajectories. Again, this suggests that attending to a moving object creates an object file in which object identity and spatiotemporal parameters are closely integrated.

To ensure stable percepts of objects (e.g., tolerating small changes in viewpoint or lighting), the functional links or object files need to be persistent over time. Indeed, behavioral research suggests that object files outlive the events they represent by several seconds and that they affect subsequent behavior in a systematic fashion [8,9]. For example, if subjects respond to one feature (e.g., shape) of a two-dimensional stimulus (e.g., varying in shape and location), they respond faster and more accurately if the two stimulus features both repeat or both alternate, than if one feature repeats while the other alternates [9-14]. Consistent with the notion of object files, this finding suggests that processing an object binds its features such that if one or all of these features are encountered again, the whole object file is retrieved. If this involves reactivation of a feature that mismatches with features of the present object (which happens when one feature repeats and another alternates), performance is impaired because of the conflict between retrieved and perceptually available features and/or because the old associations need to be deconstructed [14]). Note that the task described here did not require participants to integrate features; therefore the obtained effects provide a relatively pure measure of automatic, implicit integration processes, free of particular task-dependent strategies [15].

**Figure 1:**
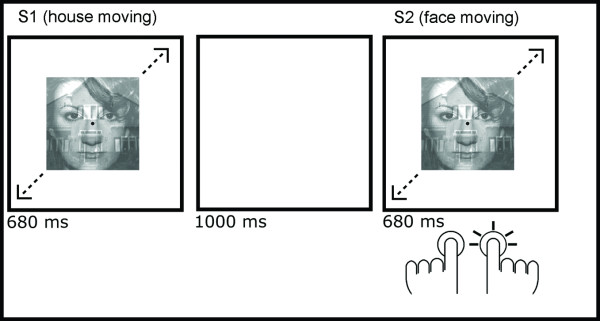
**An example trial**. On each trial two face-house compound stimuli (S1 and S2) were presented. Either the face or the house moved, in a left-up right-down or right-up left-down oscillatory fashion. Participants were instructed to watch S1 and to give a two-choice response to the direction of motion in S2, irrespective of the object that moved.

Automatic retrieval of object files has the theoretically interesting property of mimicking several effects that are often attributed to executive control processes. For example, there is evidence that at least substantial portions of the flanker-compatibility effect [16], the Simon effect [17], inhibition of return [18], and negative priming [19] are actually produced by the impact of object files formed in the previous trial. However, the neural mechanisms underlying the hypothesized object-file retrieval are unknown and direct demonstrations that feature repetition actually induces the retrieval of corresponding object files are lacking. Accordingly, the present fMRI study was designed to test whether reviewing a particular stimulus feature reactivates the features of the object it previously accompanied. The features/objects that we used to address this question were motion, faces, and houses, which, as noted above, activate distinguishable regions of the occipitotemporal cortex [7]. These stimuli have been shown to integrate in a similar way as more basic features such as location and color [20]. As in previous studies [9], participants were presented with two stimuli: A task-irrelevant prime (S1) and a probe (S2). Both stimuli consisted of blended pictures of a face and a house. On each trial, either the face or the house moved in one of two possible directions and participants were instructed to respond as quickly as possible to the direction of the moving object in S2. Thus, each stimulus consisted of two features (motion direction and moving object) that were orthogonally repeated or alternated between S1 and S2 (Figure 1).

We expected to obtain the standard behavioral result: Repeating the motion direction and the moving object, or alternating both, should yield better performance than repeating one feature and alternating the other. The fMRI measures were used to test whether this pattern actually reflects object-file retrieval. In particular, our approach was to use activity in the FFA and PPA as an effective index of the degree to which the task-relevant stimulus feature (motion direction in S2) reactivated the task-irrelevant feature (moving object) it accompanied in S1. Thus, we examined whether repeating the motion direction reactivated the object (face or house) that moved in this direction in S1. Diagnostic for this reactivation effect are conditions in which the moving object changes (e.g., if a house moved in S1 but a face moved in S2): Repeating the motion direction in S2 should tend to reactivate the representation of the house that moved in this direction in S1, which should lead to a greater activation of the PPA than if motion direction alternates.

## Methods

### Participants

Fourteen healthy, young undergraduate students volunteered in exchange for course credit or money.

### Experimental protocols

Each stimulus was composed by transparently superimposing one of eight grayscale front-view photographs of male (4) and female (4) faces on one of eight grayscale photographs of houses, following O'Craven et al. [7]. The images were cropped to fit a square size (10° by 10°) and adjusted to assure the same average luminance. Either the face or the house oscillated in a straight path on one of two possible non-cardinal directions (left-up/right-down vs. right-up/left-down), while total size of the combined images remained the same. The maximal displacement caused by the motion was less than 10% of the size of the image. The moving image oscillated 2 cycles with a constant speed of 9° per second.

A trial started with a face-house compound stimulus (S1), randomly selected from all possible combinations of identity of the face and the house, direction of the motion, and the object that moved. Following the presentation of S1 for 680 ms, a black screen was presented for 1000 ms. Then, a second face-house compound stimulus (S2) was presented, in which the identity of the face and the house were repeated from S1. Both the direction of the motion and the object that moved could be the repeated or alternated between S1 and S2. Participants were instructed to watch S1 and make a speeded left-right key press to the direction of the motion of S2, disregarding the identity of the moving object. S2 was followed by a fixation circle (0.5°), which remained on the screen for a randomly chosen duration between 1000–2500 ms, varied in 100-ms steps. After every seven trials, a fixation circle was presented for ten seconds. The experiment consisted of a total of 182 trials and 26 ten-second rest periods. At the start, halfway, and at the end of the experimental run, a fixation circle with a duration of 30 seconds was presented to provide a stable baseline measure.

The experimental run was followed by a localizer run that we used to identify each participant's face-selective and house-selective regions of interest (ROIs). This run consisted of a series of blocks in which either stationary grayscale images of houses, faces or fixation circles were presented, of the same size and luminance as the compound images in the experimental run. The images of houses and faces were presented for 680 ms, followed by a 320-ms black screen. A total of 24 images were presented per block. In the fixation-circle block, a fixation circle was presented for 24 seconds. Each house block and each face block was repeated three times and these blocks were interleaved with the fixation-circle blocks.

### Image acquisition

Images were recorded with a Philips Achieva 3-T MR scanner (Philips Medical Systems, Best, The Netherlands). Functional images were acquired using a SENSE parallel imaging gradient echo EPI sequence of 38 axial slices (resolution = 2.75 mm3 isotropic; repetition time [TR] = 2211 ms; echo time [TE] = 30 ms; flip angle = 80°; field of view = 220 mm; matrix = 80 × 80). During the experimental run, lasting 21.5 minutes, 580 volumes were collected. During the localizer run, lasting 7 minutes, 190 volumes were collected. A T1-weighted structural image (MPRAGE; 1.2 mm3 isotropic) and a high-resolution EPI scan (2 mm3 isotropic) were obtained for registration purposes.

### Image analyses

MRI data analysis was carried out using FEAT (FMRI Expert Analysis Tool) version 5.4, which is part of FSL (FMRIB's Software Library, ). Image pre-processing consisted of: slice-time correction using Fourier-space time-series phase-shifting; motion correction [21]; non-brain removal [22]; spatial smoothing using a fullwidth at half maximum Gaussian kernel of 8 mm; and mean-based intensity normalisation of all volumes. Furthermore the data were temporally high-pass filtered with a cut-off of 60 seconds to remove low-frequency artefacts using Gaussian-weighted least-squares straight line fitting. Time-series statistical analysis was carried out using FILM (FMRIB's Improved Linear Model) with local autocorrelation correction [23].

Below, a three-character code is used to summarize the experimental conditions. The first two characters indicate which objects are moving in S1 and in S2: house (H) or face (F). The third character indicates whether the direction of motion in S1 is the same as the direction of motion in S2 (=) or different (≠). For analysis of the experimental run, explanatory variables of stimulus events were created for: HH=, HH≠, HF=, HF≠, FF=, FF≠, FH=, FH≠, segregated at the onset of S2. Errors and instruction displays were modeled separately. S1 was also modeled separately, comprising all four combinations of moving object and motion direction. The hemodynamic response to each event was estimated by convolving each explanatory variable with a canonical hemodynamic response function.

The primary data analysis focused on ROIs that showed significant task-selective activity during the localizer scans. To analyze the localizer data we used a fixed-effects analysis to identify, separately for each participant, regions showing significantly (P < .001, uncorrected) greater activity during house blocks than during face blocks (PPA), and regions showing the opposite pattern (FFA). To examine the presence of the hypothesized neural reactivation effects, we computed for each of these two ROIs (PPA and FFA) and for each participant the average percent increase in fMRI signal from baseline. The resulting averaged data set allowed us to test our main hypotheses: whether motion repetition results in automatic reactivation of the previously associated moving object (a face or a house).

## Results

Mean reaction times (RTs) and percentages of errors for responses to S2 were analyzed using ANOVAs. The behavioral results replicated earlier findings with the same stimuli [20] and with other variants of the basic task [9]: RTs were slower if only one feature repeated between S1 and S2 (motion direction: 586 ms, moving object: 559 ms) compared to when both features repeated (547 ms) or alternated (556 ms). This was indicated by a significant interaction of moving-object repetition/alternation and motion-direction repetition/alternation (F[1, 13] = 25.42, p < .001). The interaction was mainly driven by an increase in RT on motion-repeat/object-alternate trials compared to complete-repetition trials (t[13] = 3.11, p < .01) and complete-alternation trials (t[13] = 5.26, p < .0005). Percentages of errors (4.6% across all task conditions) did not show an interaction of these variables (p = 0.76).

### Reactivation effect in the PPA (S1: house moving, S2: face moving)

**Figure 2:**
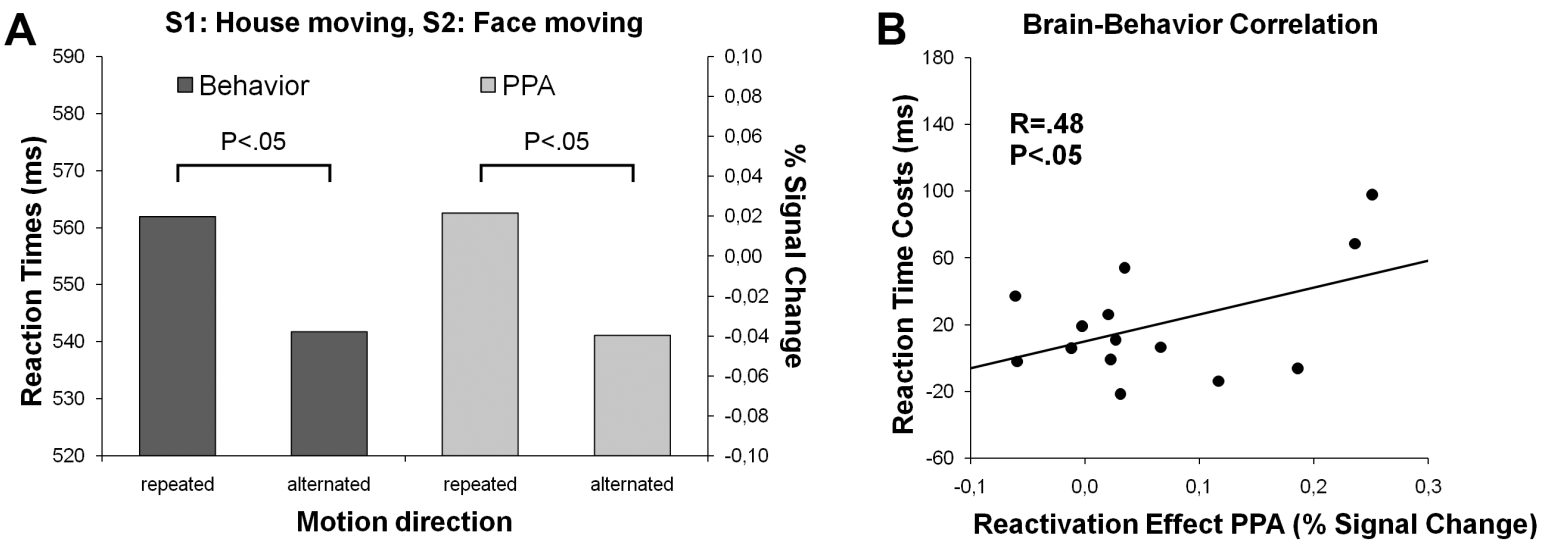
**S1: house moving, S2: face moving**. (A) Average reaction times and percent fMRI signal change in the PPA as a function of motion direction (repeated vs alternated) for trials in which a house moved in S1 and a face in S2 (i.e., alternation of moving object). Consistent with our predictions, reaction times and activity in the PPA were significantly increased when motion direction was repeated. (B) There was a significant correlation across participants between the reaction time costs and the PPA reactivation effect associated with the repetition of motion direction (in the context of an alternation of moving object).

To examine the presence of a reactivation effect in the PPA we contrasted the conditions in which the house in S1 and the face in S2 moved in the same direction versus in different directions (HF = minus HF≠). If repeating the direction of the motion reactivated the representation of the house, we would expect increased RTs and increased activation in the PPA compared to the alternating condition. The contrasts confirmed our expectations (Figure 2A): Repeating the direction of motion was associated with a reliable RT cost relative to alternating the direction of motion (562 ms vs 542 ms, F[1, 13] = 4.99, p < .05). Furthermore, the right PPA was more active on motion-repeat than on motion-alternate trials (t[13] = 2.31, p < .05), suggesting that on motion-repeat trials the presentation of the moving face in S2 reactivated the representation of the moving house in S1. Importantly, there was a significant positive correlation between the RT cost and the reactivation effect in the PPA (i.e., the difference in activation between motion-repeat and motion-alternate trials, indicating that participants with a larger reactivation effect in the PPA in general had a larger RT cost (Figure 2B).

### Reactivation effect in the FFA (S1: face moving, S2: house moving)

**Figure 3:**
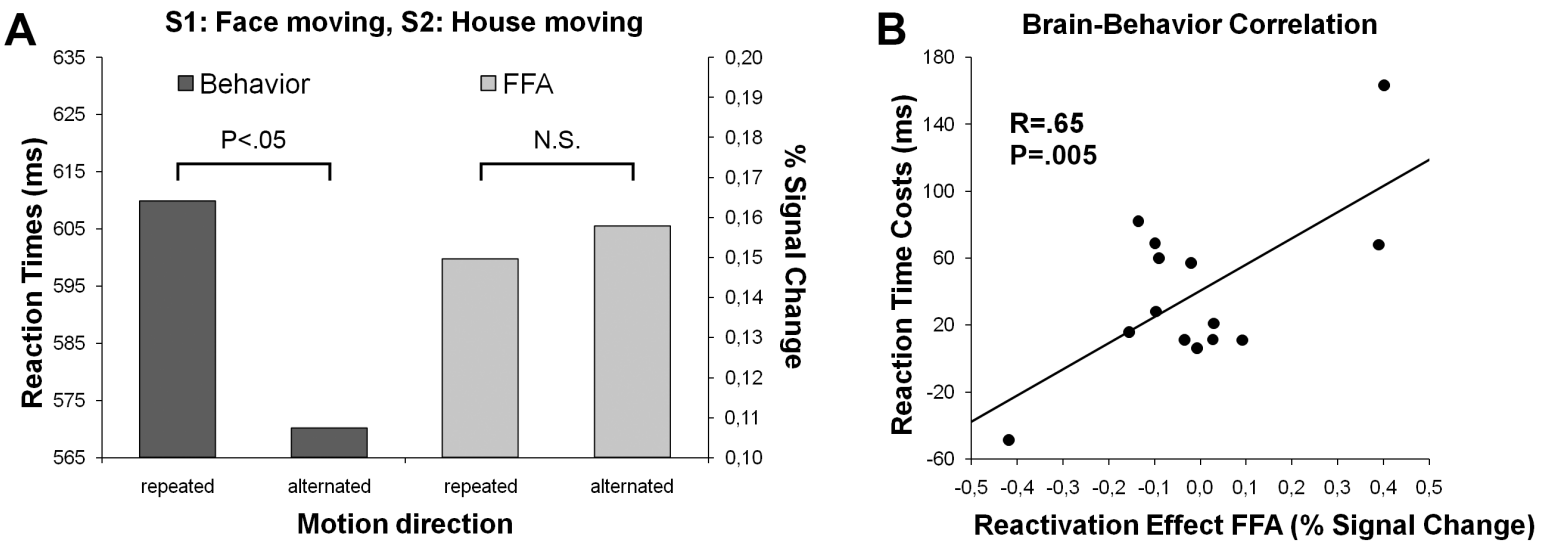
**S1: face moving, S2: house moving**. (A) Average reaction times and percent fMRI signal change in the FFA as a function of motion direction (repeated vs alternated) for trials in which a face moved in S1 and a house in S2 (i.e., alternation of moving object). Reaction times were significantly increased when motion direction was repeated. This was not the case for activity in the FFA. (B) There was a significant correlation across participants between the reaction time costs and the (nonsignificant) FFA reactivation effect associated with the repetition of motion direction (in the context of an alternation of moving object).

To examine the presence of a reactivation effect in the FFA we compared two different experimental conditions: the conditions in which the face in S1 and the house in S2 moved in the same direction versus in different directions (FH = minus FH≠). If repeating the direction of the motion reactivated the representation of the face, we would expect increased RTs and increased activation in the FFA compared to the alternating condition. These predictions were only partly confirmed (Figure 3A): Repeating the direction of motion was associated with a substantial RT cost relative to alternating the direction of motion (610 ms vs 570 ms, F[1, 13] = 8.94, p = .01). However, the FFA did not show a reactivation effect (t[13] = 0.17, p = .87). As for the PPA, there was a significant positive correlation between the reactivation effect in the FFA and the corresponding RT cost (Figure 3B). The two participants with (by far) the largest FFA reactivation effect had large RT costs, whereas the participant with (by far) the smallest FFA reactivation effect had the smallest RT costs (or rather an RT benefit). Although these observations are consistent with our hypothesis, the dominant cluster of participants did not display the predicted positive correlation, either because this correlation is not present in the hypothetical population, or because there was not sufficient range in the individual FFA reactivation effects to reveal an existing correlation.

## Discussion

The behavioral results replicated previous findings in showing that the partial repetition of stimulus features impairs performance [9,10,20]. This pattern has been attributed to the binding of feature representations upon stimulus (S1) presentation and the automatic retrieval of the whole object file if one or more features are encountered again (in S2). If this retrieval includes a feature that does not match the present stimulus, feature conflict occurs, yielding an increase in RT. In this study we investigated whether there is neural evidence for a corresponding object-file retrieval effect in brain areas representing specific stimulus features. Such evidence would provide critical support for theoretical accounts of feature binding and its consequences on subsequent information processing [10].

The fMRI data provide encouraging support for our hypothesis. The right PPA, a house-selective brain area, showed increased activation to moving faces (in S2) if a couple of seconds earlier the same direction of motion had been paired with a house (in S1), compared to when both the direction of motion and the moving object alternated. This supports the view that the presentation of a stimulus feature (a particular direction of motion) reactivates features it was previously bound with in an object file (house). The finding of a reliable positive relationship between the observed reactivation of the PPA and the partial repetition cost is consistent with the possibility that the neural reactivation effect *caused *the corresponding performance costs. Thus, neural and behavioral measures of the reactivation of the inappropriate feature (the house, when a face was actually moving) were closely correlated across participants.

The fMRI data for the FFA, a face-selective brain area, did not reveal unequivocal evidence for our hypothesis. On the one hand, the reactivation effect in this area showed the predicted positive relationship with performance costs, suggesting that repeating the motion does modulate activity in the FFA. On the other hand, however, the correlation was not clearly representative of the majority of the participants, and even though the individual RT costs were generally substantial (and larger than those associated with the PPA reactivation effect), most of the participants did not show the predicted FFA reactivation effect. At this point, we can only speculate why the FFA showed a different behavior than the PPA. For example, there is evidence that stimuli of greater biological significance, such as faces, attract more attention and induce more activation [24,25]. As a result, activation in the FFA may be less sensitive to subtle modulations like the reactivation effect.

## Conclusion

These caveats notwithstanding, the current findings are important for the vast body of behavioral research focusing on the after-effects of feature integration [8], and provide evidence concerning the neural underpinnings of the principles summarized in the Theory of Event Coding (TEC, [9]), which addresses the origin of performance costs observed in the partial repetition of features. Our findings also extend the study of O'Craven et al. [7] in providing new insights into the way the representation of multi-featured objects in visual brain areas has an impact upon reviewing those objects. In particular, our study provides the first neural evidence that the brain binds together the features of an object on a single presentation, and reactivates all the bound features when at least one of these features is encountered later on.

## Competing interests

The authors declare that they have no competing interests.

## Authors' contributions

AWK has been responsible for the designing the experimental protocol, acquiring the data, analyzing the data and writing the manuscript. LSC participated in the data acquisition. WT designed the fMRI protocol. SN, SARBR and BH participated in discussions about the data analyses. All authors contributed to, read and approved of the final manuscript.
